# Determination of Mammalian Target of Rapamycin Hyperactivation as Prognostic Factor in Well-Differentiated Neuroendocrine Tumors

**DOI:** 10.1155/2017/7872519

**Published:** 2017-10-29

**Authors:** G. Lamberti, C. Ceccarelli, N. Brighi, I. Maggio, D. Santini, C. Mosconi, C. Ricci, G. Biasco, D. Campana

**Affiliations:** ^1^Department of Experimental, Diagnostic and Specialty Medicine, S.Orsola-Malpighi University Hospital, Bologna, Italy; ^2^Department of Diagnostic and Prevention Medicine, S.Orsola-Malpighi University Hospital, Bologna, Italy; ^3^Department of Medical and Surgical Sciences, S.Orsola-Malpighi University Hospital, Bologna, Italy

## Abstract

**Purpose:**

To evaluate the role of the activation of mTOR (phosphorylated mTOR, p-mTOR) and the expression SSTR2A and IGF-1R as prognostic factor in well-differentiated neuroendocrine tumors.

**Methods:**

A retrospective study was conducted on data from patients with diagnosis of neuroendocrine tumor originated from pancreas (pNET) or gastrointestinal tract (stomach, appendix, and ileus; GI-NET) made between January 2003 and December 2004 and followed up at our institution. Archival material should be available for revision according to WHO 2010 neuroendocrine tumor classification and for p-mTOR, SSTR2A, and IGF-1R immunostaining, calculating a quantitative score (QS). We evaluated clinical, pathological, and immunohistochemistry features for association with the presence of advanced disease at diagnosis and disease relapse in patients who have undergone radical surgery.

**Results:**

Archival material from 64 patients was analyzed (37 pNETs and 27 GI-NETs). In these patients, G2 grading, low SSTR2A QS, and high p-mTOR QS were associated with advanced disease at diagnosis at multivariate analysis. Risk of recurrence in 49 patients with R0-resected tumors was higher for G2 grading, stage IIIB-IV, low IGF-1R QS, and high p-mTOR QS at univariate analysis.

**Conclusions:**

With the limits of retrospective data, activation of m-TOR is correlated with advanced disease at diagnosis and with shorter disease-free survival after R0 resection. Validation through prospective studies is needed.

## 1. Introduction

The incidence of neuroendocrine tumors (NETs) had risen over time in the last years; therefore, it is no longer considered rare tumors [1]. The increasing number of patients raised the need for prognostic and predictive factors to help in clinical management.

Prognostic factors are clinical, laboratorial, or radiological features of patient or disease which help to forecast aggressiveness in tumor behavior, thus, providing an insight into tumor biology and information to improve clinical decision making and trial design.

Neuroendocrine tumors can arise from any district throughout the body, but two-thirds of them originate from gastroenteropancreatic- (GEP-) NET district [2]. Gastroenteropancreatic- (GEP-) NET grading is defined by Ki67 proliferation index as stated in the World Health Organization (WHO) 2010 classification, so that G1, G2, and G3 are defined by Ki67 ≤ 2%, 3–20%, and >20%, respectively [3]. Stage at the time of diagnosis and grade are the main established prognostic factors in NETs. Alongside with the few validated prognostic factors available, more have been investigated [4–6].

Somatostatin receptor has 5 subtypes (SSTR1-5) that are widely and variably expressed on NETs. Targeting SSTRs is the mainstay of treatment of GEP-NETs, both for control of syndrome and for tumor growth [7, 8]. Beyond its predictive value for peptide receptor radionuclide therapy [9], expression of SSTRs has been investigated and found correlated with favorable prognosis in GEP-NETs [10, 11].

The activation of insulin-like growth factor 1 receptor (IGF-1R) promotes cell growth, neoangiogenesis, and invasiveness [12]. The IGF-1R was found to be expressed in various GEP-NETs [13]. Its prognostic significance is unclear [13, 14].

After p53, the second most mutated pathway in tumors is PI3K pathway [15], which has a relevant role in NETs [16, 17]. The mammalian target of rapamycin (mTOR) is a protein downstream in PI3K pathway, and its gene is mutated in 15% of pancreatic NETs (pNETs) [18]. Hyperactivation of PI3K pathway may be highlighted by means of immunohistochemistry staining of the phosphorylated proteins of the pathway. Being mTOR downstream in the pathway, phosphorylated mTOR protein (p-mTOR) can be used to disclose pathway activation.

The presence of p-mTOR on tumor specimens has been studied for both prognostic [19–21] and predictive value [22] with opposing results.

We ran a retrospective study in our institution to evaluate whether presence of p-mTOR was associated with more aggressive NETs both at diagnosis and during follow-up.

## 2. Materials and Methods

### 2.1. Patients

We retrospectively analyzed specimens from consecutive patients who received diagnosis of well-differentiated GEP-NET at our institution between January 2003 and December 2004.

Inclusion criteria were as follows:

- Diagnosis made on surgical or biopsy specimen according to WHO 2000 classification
- Enough and adequate material to revise diagnosis according to WHO 2010 and to do the immunostaining
- Follow-up conducted at our institution

We excluded patients with diagnosis of poorly differentiated endocrine carcinoma (PDEC) GEP-NET according to WHO 2000 classification, with diagnosis of NEC according to WHO 2010 classification after revision, or without material for revision or immunostaining.

Patients with multiple endocrine neoplasia and Von Hippel-Lindau syndromes were also excluded.

We retrieved and registered data about age and diagnosis; gender; date of diagnosis; sample on which diagnosis was made (biopsy or surgery); primary NET site (pancreas, stomach, ileum, colon, and appendix); presence and type of associated syndrome (carcinoid, insulinoma, glucagonoma, and Zollinger-Ellison); presence MEN-1 diagnosis; histopathological characteristics at diagnosis (including stage, presence of lymph node metastasis, liver metastasis, or other distant metastases and grading); first-line treatment (surgical, regional, and systemic/medical); type of surgery if any; outcome of surgery (R0: no residual disease at histological examination, R1: microscopic residue, R2: macroscopic residue); medical treatment (SSA: somatostatin analogues, PRRT: peptide receptor radionuclide therapy, CHT: chemotherapy, TKI: tyrosine-kinase inhibitor); duration of follow-up; recurrence/progression occurrence; and death.

Patients were followed up at our institution by CT scan with contrast mean every 6 months or less if clinically indicated. When CT scan was not indicated (e.g., iodine contrast mean anaphylaxis), MRI scan was performed. CT scan and MRI findings were further investigated by means of 68Ga-DOTA-NOC positron emission tomography (PET) or endoscopic ultrasonography (EUS) when appropriate.

### 2.2. Immunostaining

Immunohistochemical analysis (IHC) was performed on formalin-fixed-paraffin-embedded serial sections collected on precharged slides (Tom-11, Matsunami Glass Ind. Ltd, Japan). Sections were dewaxed, rehydrated, and retrieved using a citrate buffer solution pH 6.0 (40 min at 98.5°C) or a Tris-EDTA buffer solution pH 9.0 (20 min at 98°C) in a circulating water bath for anti-p-mTOR (Ser2448) clone 49F9 rabbit monoclonal antibody (Cell Signaling Technology Inc., USA) diluted 1 : 80 or anti-IGF-1R goat polyclonal antibody (R&D Systems, USA) diluted 1 : 100, respectively. Endogenous peroxidase activity was quenched with a methanol/H_2_O_2_ 1.5% solution (20 min at room temperature (RT)). Primary antibodies were incubated overnight at RT in a humid chamber and then processed with a nonbiotin peroxidase-amplified system (Novolink, Novocastra Lab, UK) according to the manufacturer's instructions. A rabbit antigoat (H + L) antibody (Vector Lab, USA) diluted 1 : 500 was used for anti-IGF-1R polyclonal antibody before Novolink administration. The immunological reaction was visualized with a 3-3′-diaminobenzidine tetrahydrochloride/H_2_O_2_ solution. Sections were then counterstained in Harris hematoxylin and dehydrated and mounted in Bio-Mount (Bio-Optica, Milan, Italy). Antisomatostatin 2A receptor (SSTR2A) immunostaining was performed on Ventana Benchmark Ultra immunostainer. Briefly, sections were retrieved on-board with ultraCC2 buffer pH 6.0 (24 min at 95°C), quenched for endogenous peroxidase activity, and incubated with anti-SSTR2A rabbit monoclonal antibody clone UMB-1 (Epitomics, Abcam plc., UK) diluted 1 : 300 (16 min at 37°C). The reaction was visualized using the OptiView DAB detection system.

### 2.3. Immunostaining Evaluation

The entire neoplastic population was evaluated at 200x and the quantitative score (QS) was calculated. For each microscopic field, immunostained cells were scored according to both positive percentage (*P*) and staining intensity (*I*).

The “*P*” score was 0 if there was <1% of stained cells, 1 if 1–25%, 2 if 25%–50%, 3 if 50%–75%, and 4 if >75%.

The “*I*” score was calculated upon intensity of staining: weak = 1, intermediate = 2, and strong = 3.

The final QS used for analyses was the numerical product of *P* score and *I* score (*P* × *I* = QS).

### 2.4. Statistical Analysis

All data were prospectively collected at our center. A computerized data sheet was created, and data regarding demographic, clinical, and pathological features were retrospectively analyzed. The histological specimens were examined by an experienced pathologist. Tumors were classified according to the 2010 WHO classification (gastroenteropancreatic neuroendocrine tumors) and the ENETS grading system (Rindi et al. 2006; Rindi et al. 2007). The Ki67 proliferation index was expressed as a percentage based on the count of Ki67-positive cells in 2000 tumor cells in the areas of the highest immunostaining using the MIB1 antibody (DBA, Milan, Italy). Analysis of the predictive factors of metastatic disease at diagnosis was carried out by univariate and multivariate analysis using logistic regression. Predictive factors were expressed as odds ratio (OR) [95% confidence interval (CI)]. The multivariate model was constructed using the forward stepwise method after including all variables. Disease-free survival (DFS) was defined as the interval between radical surgery and the time of disease relapse. Disease-free survival was measured using the Kaplan-Meier method, and the results were compared using the log-rank test. Analysis of the predictive risk factors for disease relapse was carried out by univariate and multivariate analysis using the Cox proportional hazard method. Risk factors were expressed as hazard ratios (HR) [95% confidence interval (CI)]. The multivariate model was constructed using the forward stepwise method after including all variables. All analyses carried out for predictive and risk factors are listed in the tables. The area under the receiver-operating-characteristic (ROC) curve was evaluated to determine the accuracy of the p-mTOR, SSTR2A, and IGF-1R QS in predicting advanced disease at diagnosis. The best prognostic cut-off value was estimated by a maximum likelihood ratio method. The distribution of the continuous variables was reported as median, range, and interquartile range (IQR, 25th to 75th percentiles). The comparison between the subgroups was carried out using Pearson's chi-squared test (Fisher's exact test was used when appropriate) or the Mann–Whitney *U* test for continuous variables. The *p* value was considered significant when less than 0.05. The statistical analysis was carried out using a dedicated software (IBM SPSS Statistics v. 22).

## 3. Results

We selected 64 patients matching to inclusion criteria, whose characteristics are summarized in Table 1. Median age was 59 years (range 21–79), 26 were men (40.6%) and 38 were women (59.4%). Thirty-seven (57.8%) patients had pNET, while 27 (42.2%) had gastrointestinal tract NET (GI-NET: ileus, stomach, or appendix). Fourteen (21.9%) patients had associated syndrome, 9 had insulinoma syndrome, 2 had hypergastrinemia (Zollinger-Ellison syndrome (ZES)), and 3 had carcinoid syndrome due to serotonin hypersecretion.

The proliferation index was available in 63 NETs; median Ki67 was 1.8% (range 0.1–18.8) so that 39 (60.9%) were G1 NETs and 25 (39.1%) were G2 NETs, according to WHO 2010 classification.

**Figure 1:**
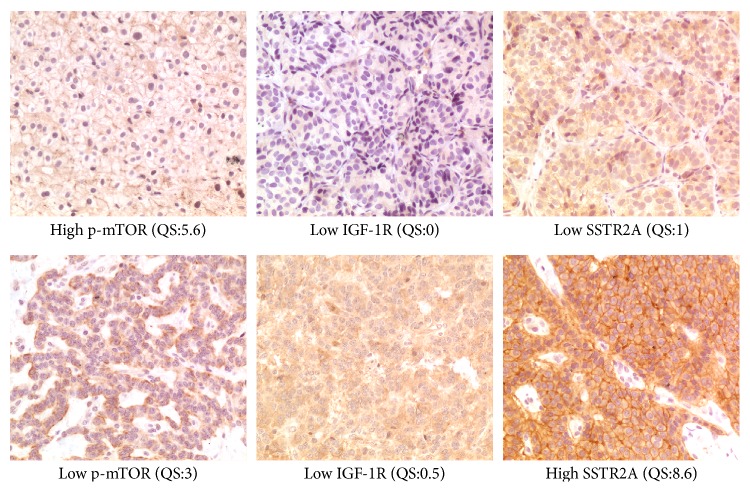
Immunostained samples (200x) and corresponding quantitative scores (QS).

Quantitative score was evaluated for SSTR2A, IGF-1R, and p-mTOR immunostaining on all samples (Figure 1).

### 3.1. p-mTOR

**Figure 2:**
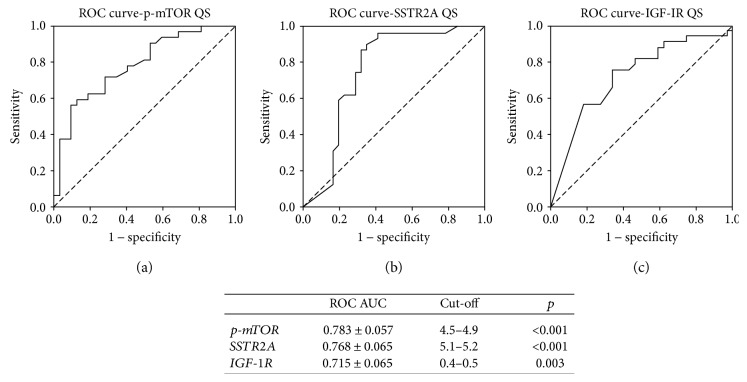
Receiver-operating curve for p-mTOR (a), SSTR2A (b), and IGF-1R (c) QSs and stage IIIB-IV at diagnosis.

As reported in Table 2, median QS (range) was 3.05 (0–9.20). The QS of p-mTOR was higher in patients without syndrome than in patients with any syndrome (median 3.45 versus 1.00, *p* = 0.033) and in GI-NETs than in pNETs (median 3.10 versus 0.80, *p* < 0.001). Moreover, p-mTOR QS was higher in stage IIIB-IV NETs than in stage I-II (median 5.0 versus 2.0, *p* < 0.001). No differences were found between male and female patients (median 3.6 versus 3.0, *p* = 0.18) and between G1 and G2 NETs (median 2.9 versus 4.0, *p* = 0.143). Receiver-operating-characteristic curves were used to determine the best cut-off ranges above which p-mTOR QS correlated with metastatic disease at diagnosis. A p-mTOR QS higher than 4.5–4.9 correlated with metastatic disease at diagnosis (AUC 0.783 ± 0.057; *p* < 0.001). Twenty-one patients (32.8%) had a high p-mTOR QS according to this cut-off (Figure 2(a)).

### 3.2. SSTR2A

As reported in Table 2, median QS (range) was 3.75 (0–11.7). The QS of SSTR2A was significantly higher in stage I-II NETs than in stage IIIB-IV (median 6.40 versus 2.10, *gem*). No differences were found between male and female patients (median 2.7 versus 4.6, *p* = 0.593), syndromic and nonsyndromic patients (median 2.4 versus 4.3, *p* = 0.116), pNETs and GI-NETs (median 4.8 versus 3.0, *p* = 0.801), and G1 and G2 NETs (median 4.3 versus 2.8, *p* = 0.158). Receiver-operating-characteristic curves were used to determine the best cut-off ranges below which SSTR2A QS correlated with metastatic disease at diagnosis. Receiver-operating-characteristics curve showed that a SSTR2A QS lower than 5.1–5.2 correlated with metastatic disease at diagnosis (AUC 0.768 ± 0.065; *p* < 0.001). According to this cut-off, 38 patients (59.4%) had a low SSTR2A QS (Figure 2(b)).

### 3.3. IGF-1R

As reported in Table 2, IGF-1R QS (range) was 0.25 (0–8.0). The QS of IGF-1R was higher in NET G1 than in G2 (median 0.9 versus 0, *p* = 0.005) and in stage I-II than in IIIB-IV (median 1.10 versus 0, *p* = 0.002, resp.). No differences were found between male and female patients (median 0.3 versus 0.3, *p* = 0.496), syndromic and nonsyndromic patients (median 0.5 versus 0.3, *p* = 0.707), and pNETs and GI-NETs (median 0.5 versus 0.2, *p* = 0.276). Receiver-operating-characteristic curves were used to determine the best cut-off ranges below which IGF-1R QS correlated with metastatic disease at diagnosis. A IGF-1R QS lower than 0.4–0.5 correlated with metastatic disease at diagnosis (AUC 0.715 ± 0.065; *p* = 0.003). Using this cut-off, IGF-1R QS was low in 35 (54.7%) specimens (Figure 2(c)).

The univariate analysis showed a statistically significant correlation between advanced NET at diagnosis (i.e., stage IIIB-IV) and G2 grading, low SSTR2A QS, low IGFR-1R QS, and high p-mTOR QSs. The analysis did not show any correlation for gender (*p* = 0.611), presence of syndrome (*p* = 0.546), and primary NET site (*p* = 0.079). A multivariate analysis confirmed that there was a statistically significant correlation between G2 grading (OR 77.0, CI 95% 5.6–1064.2, *p* = 0.001), low SSTR2A QS (OR 121.3, CI 95% 6.8–2174.9, *p* = .001), and high p-mTOR QS (OR 81.0, CI 95% 4.9–1353.7, *p* = 0.002) and stage IIIB-IV disease at diagnosis (Table 3).

### 3.4. Disease-Free Survival Analysis

Follow-up data were available for 49/64 patients (76.6%). Median age was 59 years (range 30–79), 22 were males (44.9%) and 27 were females (55.1%). A pNET was the primary tumor in 36 (73.5%), while GI-NET was in 13 (26.5%). Twenty-eight patients (57.1%) had a G1 NET and 21 were G2 (42.9%) while 27 (55.1%) had metastatic disease at diagnosis (stage IIIB-IV) and 22 (44.9%) had localized disease (stage I-II).

The SSTR2A QS was high in 17 of these patients with available follow-up data (34.7%), IGF-1R QS was high in 20 (40.8%), and p-mTOR QS was high in 17 of them (34.7%).

Of these patients, 46 patients (93.9%) underwent surgery after diagnosis; the outcome of surgery was absence of macroscopically evident disease and free margins at histological analysis (R0) in 40 patients (87%), while there was evidence of leftover malignant tissue (R2) in 6 patients (13%). When R0 resection was achieved, patients started follow-up (no adjuvant therapy was administered, according to ENETS guidelines [23, 24]). Three of the 6 patients who had R2 resection received first-line treatment with SSAs, while 3 patients had PRRT followed by SSA; finally, 3 patients already had metastases at diagnosis and had PRRT followed by SSA as first-line therapy.

To evaluate whether baseline histopathological characteristics and immunostaining QS were correlated with disease recurrence, we analyzed DFS in the 40 patients who underwent R0 surgery only. In this subgroup of patients, median DFS was 69.5 months (range 4–140, IQR 41.5–94.0).

**Figure 3:**
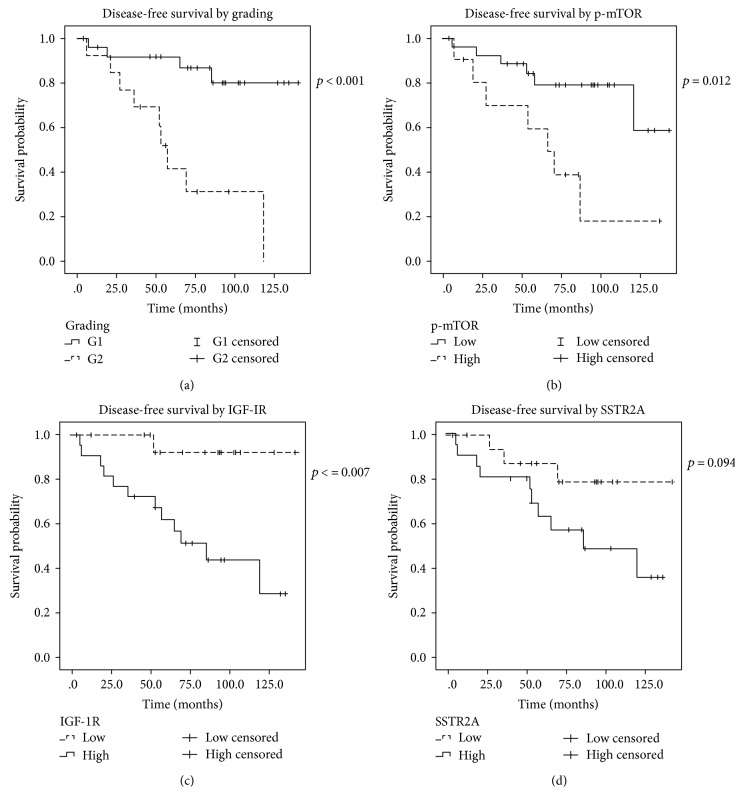
Disease-free survival curves after radical surgical resection at diagnosis according to grading (a), p-mTOR QS (b), IGF-1R (c), and SSTR2A (d).

A statistically significant difference was present in the DFS curves when stratified by grading, stage at diagnosis, IGF-1R, and p-mTOR, while there was no difference when stratified by gender (*p* = 0.98), presence of syndrome (*p* = 0.258), site of primary NET (pNET versus GI-NET, *p* = 0.268), and SSTR2A QS (low versus high, median 118 months versus not reached, *p* = 0.094; Figure 3(d)). In particular, DFS was shorter in G2 than in G1 (median 57 months versus not reached, *p* < 0.001; Figure 3(a)), in stage IIIB-IV at diagnosis than in stage I-II (median 55 months versus not reached, *p* < 0.001), in low IGF-1R QS than in high IGF-1R QS (median 85 months versus not reached, *p* = 0.007; Figure 3(c)), and in high p-mTOR QS than in low p-mTOR QS (median 69 months versus not reached, *p* = 0.012; Figure 3(b)).

### 3.5. Risk Factors for Disease Relapse

Relationship between disease relapse and gender, presence of any syndrome, primary site of NET, grading, stage at diagnosis, SSTR2A QS, IGF-1R QS, and p-mTOR QS was investigated.

As shown in Table 4, at the univariate analysis, G2 NETs had a higher risk of recurrence than G1 NETs (HR 6.578, CI 95% 1.98–21.82, *p* = 0.002), stage IIIB-IV NETs at diagnosis had a higher risk of recurrence than stage I-II after R0 surgery (HR 105.30, CI 95% 1.04–10718.10, *p* = 0.048), low IGF-1R QS had a higher risk of recurrence than high IGF-1R QS (HR 9.893, CI 95% 1.29–76.18, *p* = 0.028), and high p-mTOR QS had a higher risk of recurrence than low p-mTOR QS (HR 3.70, CI 95% 1.23–11.10, *p* = 0.02).

No difference in risk for disease relapse was highlighted in respect to gender (*p* = 0.98), presence of syndrome (*p* = 0.282), primary site of NET (*p* = 0.276), and SSTR2A QS (*p* = 0.11).

Multivariate analysis was performed as well. However, no variables affected DFS in a statically significant manner, but stage at diagnosis (HR 105.154, CI 95% 2.1–3099.1, *p* < 0.000001).

## 4. Discussion

Our retrospective study shows that activation of mTOR, as measured by means of immunostaining of p-mTOR, in radically operated pNET and GI-NET is correlated with the presence of more advanced disease at diagnosis and is a negative prognostic factor in respect to relapse and disease-free survival.

As shown by Jiao et al. in 2011 [18] and more recently by Scarpa et al. [25], about 15% of pNETs harbors mutations in the mTOR pathway. However, it does not completely explain the clinical benefit observed in RADIANT phase III clinical trials of everolimus in advanced pNET [26] and lung and GI-NET [27]; especially in the RADIANT-3 trial, mTOR mutation rate is higher than the observed response rate (5%) but lower than disease control rate (about 78%). Efforts have been made to find out whether there is any prognostic value of alterations in the mTOR pathway as we did in our study, with discordant results.

In particular, Qian et al. in 2010 did not observe any association between high p-mTOR and overall survival on a series of archive material from 173 NETs, including pNETs and GI-NETs. They also found no correlation between p-mTOR and disease-free survival in GI-NET patients who had their tumor radically removed [28]. Komori et al. found no relationship between p-mTOR and disease-free survival in 42 pNETs as well, even though high p-mTOR staining was associated with more aggressive tumor features, like bigger tumor dimension, higher grade, and more advanced stage at diagnosis [21]. Alongside these studies showing no association between p-mTOR and prognosis in NETs, there are some which highlighted p-mTOR as a prognostic factor. In a work by Yoon et al., for example, high p-mTOR at baseline and its increase after therapy with everolimus were positively associated with progression-free survival in a cohort of 54 advanced gastric cancer patients on a phase II study [20]. By contrast, in a phase II study on temsirolimus, another mTOR inhibitor, in 37 NEC patients, low p-mTOR after therapy correlated with longer time to progression [22]. Similar to the latter study, our data showed an inverse correlation between mTOR activation and prognosis in NET patients. In fact, high p-mTOR QS is associated with poorer prognosis in our study, as it correlated with the presence of metastatic disease at diagnosis and a shorter disease-free survival after radical resection.

In our study, we also characterized somatostatin receptor 2A (SSTR2A) and insulin-like growth factor I receptor (IGF-1R) expression to investigate their prognostic value and reduce potential contribution by confounding factors. This was done following various reports in literature about prognostic significance of both SSTR2A [10, 11] and IGF-1R [14] as prognostic factors in NETs, even if with discordant results.

Our results were basically discordant with those previously obtained. Song et al. analyzed 199 surgically removed G1-3 pNETs for their SSTRs expression (form type 1 to type 5) and its clinical significance. They found an increased expression of SSTR2 in functioning NETs as compared to nonfunctioning NETs and no association with lymph node metastases at diagnosis, as opposite to our study where there was no statistically significant correlation between SSTR2 expression and presence of syndrome (*p* = 0.116) but low SSTR2A QS was associated with stage IIB-IV disease at diagnosis [11].

Similarly, Kim et al. retrospectively analyzed 247 GEP-NETs archival material for SSTRs expression. They showed that SSTR2 expression was associated with better differentiation and no lymph node involvement [10], as opposite to our results, but also to a lower pathological stage, just like in our analysis, in which low SSTR2A QS was associated with stage IIIB-IV NET at diagnosis.

On the side of prognostic value, both studies found a positive correlation between SSTR positivity and outcome. In the first study reported, SSTR2 and 5 positive NETs had better outcome than SSTR2 and 5 negative NETs, and in G2 NETs, any SSTR positivity was associated with better prognosis. In the second study, positive immunostaining for SSTR2 was associated with better overall survival. In our study, however, SSTR2A did not show any prognostic influence in DFS (*p* = 0.11).

To our knowledge, the only study addressing the prognostic role of IGF-1R in NETs patients was led by Furukawa et al. who investigated IGF and IGF-1R expression in 54 gastrinomas by means of RT-PCR for mRNA and IHC. They found that “focal” pattern of IHC staining (4/31 specimens) was associated with aggressive growth, presence of liver metastases, and disease relapse after surgery in respect to diffuse staining pattern (27/31) [14]. These could be considered similar to our results in which a low IGF-1R QS was associated with stage IIIB-IV disease at diagnosis and with a shorter DFS after radical resection.

Our analysis took into consideration all SSTR2A, p-mTOR, and IGF-1R QSs. The better stratification possibly derived by this analysis may explain why our study found a statistically significant prognostic value for p-mTOR, as opposite to other analogue studies. Nevertheless, multivariate analysis did not confirm p-mTOR prognostic value. This finding may be due to dependence between stage and p-mTOR QS in determining prognosis, or statistically powerful stage influence, which prevailed and did not let any other factor come to light, including grading, which is a known prognostic factor in NETs.

Limitations of our study are mainly its retrospective nature and inhomogeneity of sample. Both are main due to NETs known rarity. It makes difficult to undertake prospective studies with proper sample size, so the retrospective design is the most cost-effective study able to give some clinically useful information with reasonable costs and running times, even if they can include biases, mainly selection biases. To minimize this risk, we chose consecutive patients who came at our institution with enough biological material to undergo revision and immunostaining. The counterpart of this approach was the need to include different types of well-differentiated NETs to reduce the period of selection and increase sample size.

In conclusion, our study showed that hyperactivation of mTOR is associated with more advanced disease at diagnosis in patients affected by NET originated from pancreas or gastrointestinal tract. Moreover, our results showed that hyperactivation of mTOR is a prognostic factor for a shorter disease-free survival after radically removed NET from pancreas or gastrointestinal tract.

## Figures and Tables

**Table 1:** Demographics of study population.

Primary site	Pancreas	Gastrointestinal tract (ileum, stomach, and appendix)	Total
Number of pts	37	27	64 (M: 26)
Age median (range)	59.5 (30–79)	57 (21–78)	59 (21–79)
Syndrome	9 insulinoma	—	9 insulinoma
	1 carcinoid syndrome	2 carcinoid syndrome	3 carcinoid syndrome
	1 ZES	1 ZES	2 ZES
Stage	I-II: 22	I-II: 10	I-II: 32
	IIIB-IV: 15	IIIB-IV: 17	IIIB-IV: 32
Ki67 median (range)	1.8% (0.1–18.8%)	2.3% (0.5–12.4%)	1.8% (0.1–18.8%)
Grading	G1: 21 (56.8%)	G1: 18 (66.7%)	G1: 39 (60.9%)
	G2: 16 (43.2%)	G2: 9 (33.3%)	G2: 25 (39.1%)
Follow-up	Median 69.5 months (range: 4–140 months)		

ZES: Zollinger-Ellison syndrome.

**Table 2:** Subgroup comparisons of SSTR2A, IGF-1R, and p-mTOR QSs.

		SSTR2A QS		IGF-1R QS		p-mTOR QS	
		Median (IQR)	*p*	Median (IQR)	*p*	Median (IQR)	*p*
Gender	F	4.6 (1.0–6.4)	0.593	0.3 (0.0–3.2)	0.496	3.0 (0.8–4.9)	0.180
	M	2.7 (1.1–7.2)		0.3 (0.0–1.8)		3.6 (1.1–8.5)	
Syndrome	Yes	2.4 (0.0–6.0)	0.116	0.5 (0.0–3.2)	0.707	1.0 (0.0–4.9)	*0.033*
	No	4.3 (1.3–7.2)		0.3 (0.0–2.3)		3.5 (1.9–6.7)	
Primary	Pancreas	4.8 (1.0–6.5)	0.801	0.5 (0.0–2.9)	0.276	2.0 (0.8–3.4)	*<0.001*
	GI	3.0 (1.1–6.4)		0.2 (0.0–1.8)		6.7 (3.1–8.8)	
Stage	I-II	6.4 (3.0–8.0)	*<0.001*	1.1 (0.1–3.3)	*0.002*	2.0 (0.3–3.6)	*<0.001*
	IIIB-IV	2.1 (0.6–4.2)		0.0 (0.0–0.7)		5.0 (2.7–8.5)	
Grading	G1	4.3 (1.0–7.6)	0.158	0.9 (0.0–3.2)	*0.005*	2.9 (0.6–5.6)	0.143
	G2	2.8 (0.8–5.6)		0.0 (0.0–0.4)		4.0 (2.0–6.1)	

SSTR2A: somatostatin receptor 2A; IGF-1R: insulin-like growth factor-1 receptor; p-mTOR: phosphorylated mammalian target of rapamycin; QS: quantitative score; IQR: interquartile range; GI: gastrointestinal.

**Table 3:** Association with metastatic disease at diagnosis (stage IIIB-IV) according to log-rank regression test.

Variable	Univariate			Multivariate		
	OR	CI 95%	*p*	OR	CI 95%	*p*
Male gender	1.296	0.5–3.5	0.611			
No syndrome	1.444	0.4–4.8	0.546			
GI-NET	2.493	0.9–6.9	0.079			ns
Grading G2	13.364	3.7–47.9	*<0.001*	77.025	5.6–1064.2	*0.001*
Low SSTR2A QS	15.400	4.3–55.8	*<0.001*	121.342	6.8–2174.9	*0.001*
Low IGF-1R QS	5.727	1.9–16.9	*0.002*			ns
High p-mTOR QS	12.429	3.1–49.3	*<0.001*	80.986	4.9–1353.7	0.002

OR: odds ratio; CI: confidence interval; ns: not statistically significant; GI-NET: gastrointestinal primary NET (versus pancreatic NET); SSTR2A: somatostatin receptor 2A; IGF-1R: insulin-like growth factor-1 receptor; p-mTOR: phosphorylated mammalian target of rapamycin; QS: quantitative score.

**Table 4:** Risk of disease recurrence according to Cox regression test.

Variable	Univariate			Multivariate		
	HR	CI 95%	*p*	HR	CI 95%	*p*
Male gender	1.014	0.3–3.1	0.98			
No syndrome	3.064	0.4–23.6	0.282			
GI-NET	1.874	0.6–5.8	0.276			
Grading G2	6.578	2.0–21.8	*0.002*			ns
Stage IIIB-IV	105.304	1.0–10,718-1	*0.048*	105.154	2.1–3099.1	*<0.000001*
Low SSTR2A QS	2.886	0.8–10.6	0.11			
Low IGF-1R QS	9.893	1.3–76.2	*0.028*			ns
High p-mTOR QS	3.700	1.2–11.1	*0.02*			ns

HR: hazard ratio; CI: confidence interval; ns: not statistically significant; GI-NET: gastrointestinal primary NET (versus pancreatic NET); SSTR2A: somatostatin receptor 2A; IGF-1R: insulin-like growth factor-1 receptor; p-mTOR: phosphorylated mammalian target of rapamycin; QS: quantitative score.
